# Supplementary material for: Rosmarinic Acid Exhibits a Lipid-Lowering Effect by Modulating the Expression of Reverse Cholesterol Transporters and Lipid Metabolism in High-Fat Diet-Fed Mice
Source: Biomolecules. 2021 Oct 6;11(10):1470. doi: 10.3390/biom11101470 (PMC8533102; doi:10.3390/biom11101470)
Supplement: Supplementary file 1 [file biomolecules-11-01470-s001.zip › biomolecules-1393316-supplementary.pdf]

<Supplementary Materials: Figure S1>

Raw images for western blotting

Figure 3A

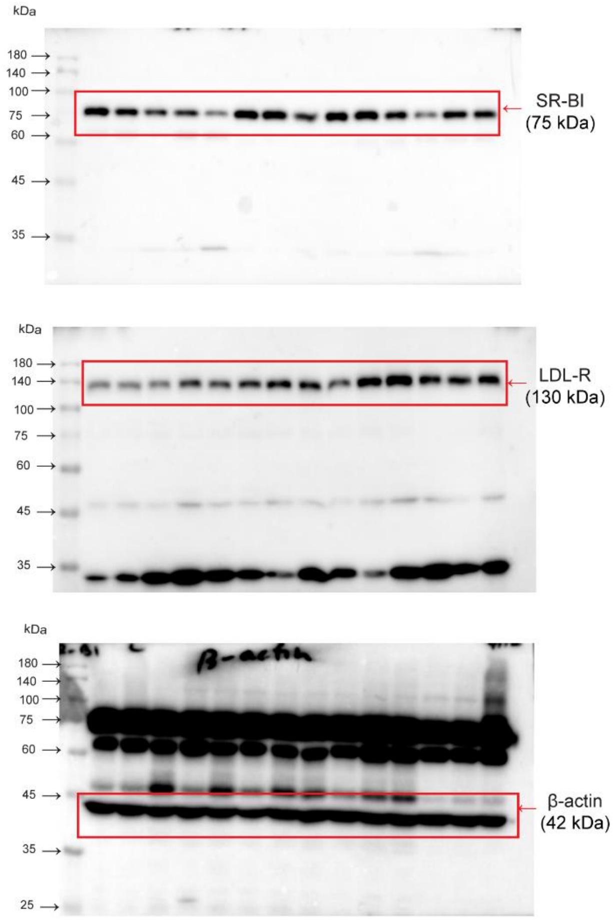

Raw images for western blotting

Figure 4A

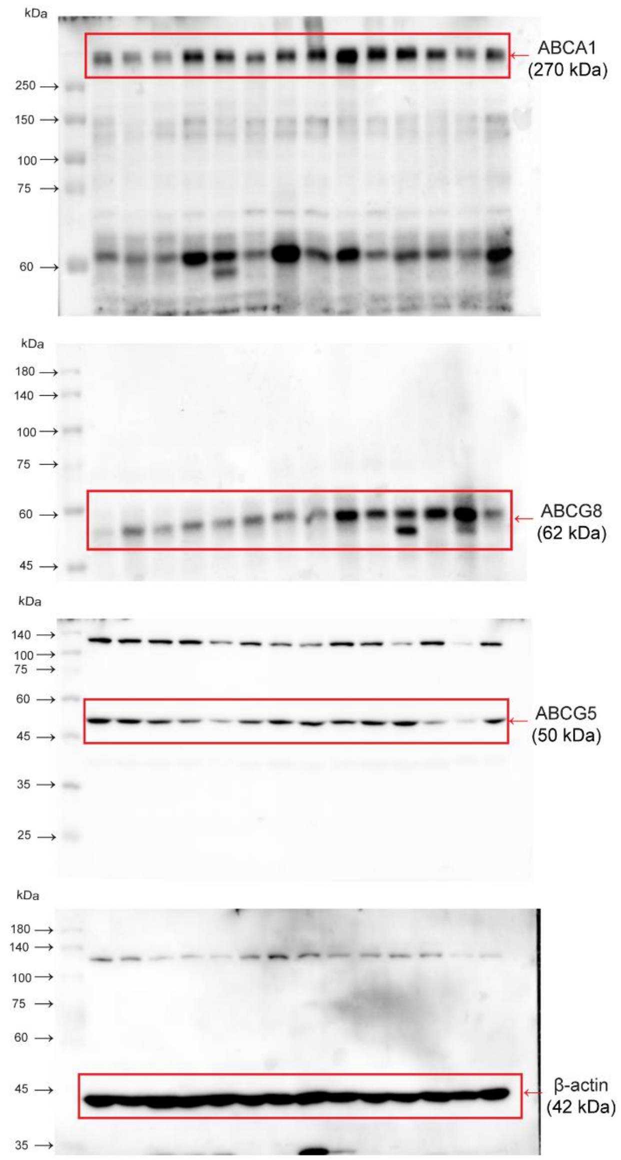

Figure 4E

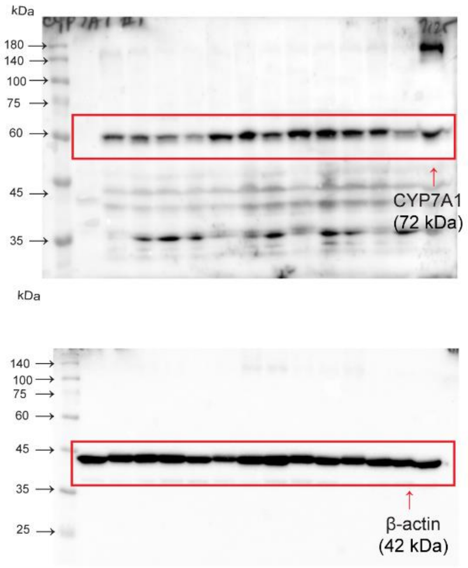

Raw images for western blotting

Figure 5A

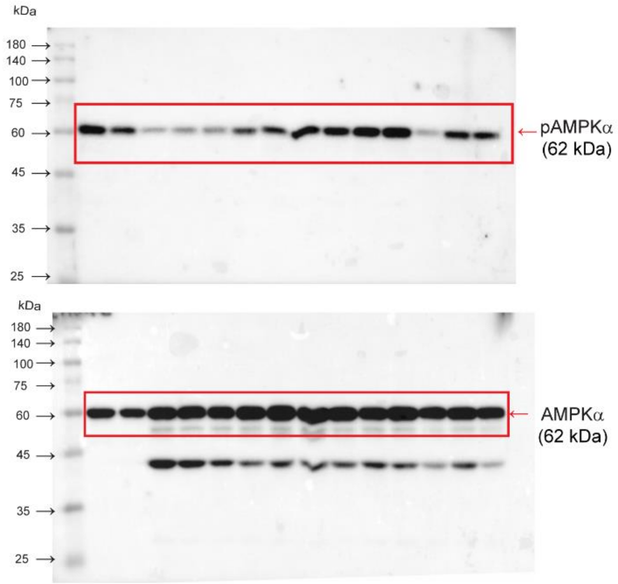

Figure 5C

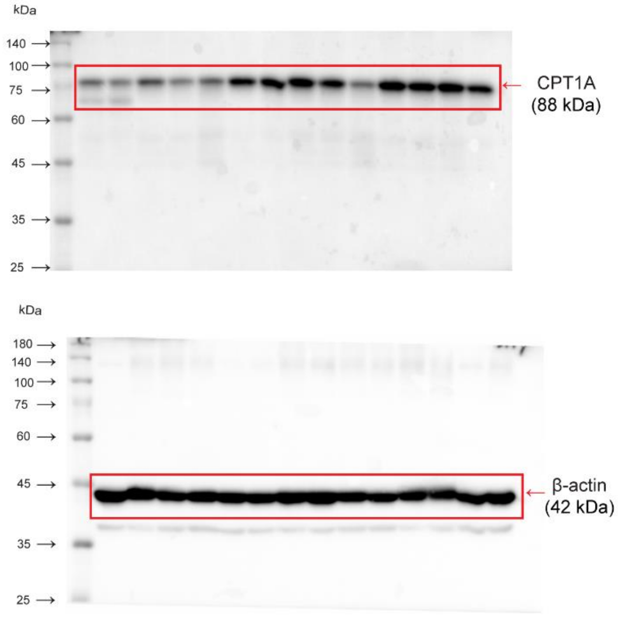

<Supplementary Materials: Tables>

<Table S1: Intensity ratios of Western blots for Figure 3B>

	Group	SR-BI	β-actin	SR-BI/β-actin	fold of control
SR-BI	Ave. of ND-1&2			0.714029795	1
	ND-1	12695.355	17467.983	0.72677853	1.017854626
	ND-2	11165.891	15922.134	0.701281059	0.982145374
	HFD-1	5714.77	17193.184	0.332385787	0.465506888
	HFD-2	8941.598	19543.719	0.457517732	0.6407544
	HFD-3	2076.527	17095.012	0.12146976	0.170118615
	HFD + RA 50-1	13535.184	16587.548	0.815984617	1.14278791
	HFD + RA 50-2	15441.477	16880.255	0.914765624	1.281130887
	HFD + RA 50-3	11908.598	20094.79	0.592621172	0.829967008
	HFD + RA 100-1	18837.719	19432.205	0.969407177	1.35765648
	HFD + RA 100-2	14024.012	16795.205	0.835000942	1.169420308
	HFD + RA 100-3	18371.305	19065.569	0.963585456	1.349503149
	HFD + ME 100-1	17381.234	21167.447	0.82113039	1.149994574
	HFD + ME 100-2	13915.184	18440.962	0.754580157	1.056790855
	HFD + ME 100-3	10439.648	20291.154	0.514492571	0.720547763
	Ave. of ND-3&4			0.746027406	1
	ND-3	15633.477	19167.79	0.815611868	1.093273331
	ND-4	11821.477	17475.941	0.676442945	0.906726669
	HFD-4	7331.062	23714.184	0.309142495	0.414384903
	HFD-5	8336.184	20079.426	0.415160473	0.556494935
	HFD-6	2843.527	20444.962	0.139082039	0.1864302
	HFD + RA 50-4	16507.598	18602.77	0.887373117	1.189464501
	HFD + RA 50-5	16619.184	19016.255	0.873946211	1.171466629
	HFD + RA 50-6	9163.355	22617.154	0.405150666	0.543077456

	**HFD + RA 100-4**	15682.355	17561.79	0.892981581	1.196982274
	**HFD + RA 100-5**	16511.184	15957.497	1.03469761	1.386943161
	**HFD + RA 100-6**	11906.77	13197.134	0.902223922	1.209371016
	**HFD + ME 100-4**	11779.648	17875.962	0.658965822	0.883299751
	**HFD + ME 100-5**	13218.184	12519.426	1.055813901	1.415248142
	**HFD + ME 100-6**	11170.062	16125.205	0.692708217	0.928529181

<Table S2: Summary of relative protein levels of Figure 3B>

SR-BI/β-actin	ND	HFD	HFD + RA 50	HFD+ RA 100	HFD + ME 100
**n=1**	1.017854626	0.465506888	1.14278791	1.35765648	1.149994574
**n=2**	0.982145374	0.6407544	1.281130887	1.169420308	1.056790855
**n=3**	1.093273331	0.170118615	0.829967008	1.349503149	0.720547763
**n=4**	0.906726669	0.414384903	1.189464501	1.196982274	0.883299751
**n=5**		0.556494935	1.171466629	1.386943161	1.415248142
**n=6**		0.1864302	0.543077456	1.209371016	0.928529181
**Mean**	**1**	**0.4056**	**1.026**	**1.278**	**1.026**
**Std. Deviation**	**0.07754**	**0.1925**	**0.2822**	**0.09632**	**0.2412**

<Table S3: Intensity ratios of Western blots for Figure 3C>

	Group	LDL-R	β-actin	LDL-R/β-actin	fold of control
LDL-R	Ave. of ND-1&2			0.345183403	1
	ND-1	7585.305	18421.548	0.411762627	1.192880723
	ND-2	5354.891	19220.426	0.278604179	0.807119277
	HFD-1	5403.891	19011.134	0.284248746	0.823471649
	HFD-2	3652.305	18172.255	0.200982487	0.582248408
	HFD-3	7464.426	17969.134	0.415402657	1.203425927
	HFD + RA 50-1	9279.134	15242.548	0.60876528	1.763599509
	HFD + RA 50-2	6595.012	19499.033	0.338222516	0.979834236
	HFD + RA 50-3	9141.012	18938.326	0.48267265	1.398307816
	HFD + RA 100-1	7067.648	19260.255	0.366955058	1.063072718
	HFD + RA 100-2	13403.305	19461.841	0.688696665	1.995161586
	HFD + RA 100-3	13333.719	19663.326	0.678100897	1.964465532
	HFD + ME 100-1	16942.548	18038.74	0.939231232	2.720962898
	HFD + ME 100-2	11409.426	17688.912	0.645004396	1.868584615
	HFD + ME 100-3	18666.497	17915.012	1.041947223	3.018532218
	Ave. of ND-3&4			0.367415404	1
	ND-3	5595.941	16122.305	0.347093111	0.944688512
	ND-4	6136.134	15825.477	0.387737697	1.055311488
	HFD-4	5906.598	16644.891	0.354859518	0.965826458
	HFD-5	10567.134	17516.598	0.603264058	1.641912811
	HFD-6	9770.548	18280.598	0.534476389	1.454692381
	HFD + RA 50-4	11569.255	18159.598	0.637087616	1.733970894
	HFD + RA 50-5	14532.669	17073.305	0.85119249	2.316703332
	HFD + RA 50-6	12882.305	19669.012	0.654954352	1.782599055

	**HFD + RA 100-4**	9283.012	10217.598	0.908531731	2.472764402
	**HFD + RA 100-5**	17920.305	16607.891	1.07902352	2.936794452
	**HFD + RA 100-6**	21180.255	18046.134	1.17367271	3.19440257
	**HFD + ME 100-4**	14242.719	16762.619	0.849671462	2.312563528
	**HFD + ME 100-5**	14025.548	15660.033	0.895626976	2.437641336
	**HFD + ME 100-6**	14280.113	17813.619	0.801640195	2.181836107

<Table S4: Summary of relative protein levels of Figure 3C>

**LDL-R/β-actin**	**ND**	**HFD**	**HFD + RA 50**	**HFD+ RA 100**	**HFD + ME 100**
n=1	1.192880723	0.823472	1.7636	1.063073	2.720963
n=2	0.807119277	0.582248	0.979834	1.995162	1.868585
n=3	1.055311	1.203426	1.398308	1.964466	3.018532
n=4	0.965826	0.965826	1.733971	2.472764	2.312564
n=5		1.641913	2.316703	2.936794	2.437641
n=6		1.454692	1.782599	3.194403	2.181836
**Mean**	**1.005**	**1.112**	**1.663**	**2.271**	**2.423**
**Std. Deviation**	**0.1618**	**0.398**	**0.4458**	**0.7702**	**0.4053**

<Table S5: Intensity ratios of Western blots for Figure 4B>

	Group	ABCA1	β-actin	ABCA1β-actin	fold of control
ABCA1	Ave. of ND-1&2			0.165389001	1
	ND-1	2827.648	13350.891	0.211794703	1.280585177
	ND-2	1746.627	14679.598	0.118983299	0.719414823
	HFD-1	2888.941	13429.477	0.215119397	1.300687444
	HFD-2	8354.891	19223.77	0.434612514	2.627819929
	HFD-3	7188.012	14679.598	0.489660003	2.960656387
	HFD + RA 50-1	5077.891	14578.82	0.348306036	2.105980646
	HFD + RA 50-2	7260.598	12833.234	0.565765262	3.420815519
	HFD + RA 50-3	5750.305	15158.134	0.379354411	2.293710031
	HFD + RA 100-1	17485.062	19553.062	0.894236514	5.406868093
	HFD + RA 100-2	13386.062	16185.891	0.827020397	5.000455838
	HFD + RA 100-3	12229.477	17896.255	0.683353975	4.131798186
	HFD + ME 100-1	7625.477	17720.548	0.430318351	2.601855915
	HFD + ME 100-2	5017.77	18841.012	0.266321682	1.610274441
	HFD + ME 100-3	7028.062	17593.255	0.399474799	2.415364961
	Ave. of ND-3&4			0.158243734	1
	ND-3	2043.627	13350.891	0.153070458	0.967308176
	ND-4	2072.062	12679.598	0.163417011	1.032691824
	HFD-4	5669.012	13429.477	0.422132001	2.667606418
	HFD-5	5944.184	18223.77	0.326177514	2.061234941
	HFD-6	6985.598	18679.598	0.373969397	2.363249317
	HFD + RA 50-4	9366.598	14578.82	0.642479844	4.060064982
	HFD + RA 50-5	6983.598	12833.234	0.54418068	3.43887663
	HFD + RA 50-6	10135.719	15158.134	0.668665352	4.225540775

	**HFD + RA 100-4**	15265.891	14553.062	1.048981376	6.628896751
	**HFD + RA 100-5**	14677.719	16185.891	0.906821812	5.7305385
	**HFD + RA 100-6**	11485.012	17896.255	0.641755049	4.055484735
	**HFD + ME 100-4**	10989.305	17720.548	0.620144761	3.918921426
	**HFD + ME 100-5**	12677.305	18841.012	0.672856904	4.252028726
	**HFD + ME 100-6**	10561.891	17593.255	0.600337516	3.793752204

<Table S6: Summary of relative protein levels of Figure 4B>

ABCA1/β-actin	ND	HFD	HFD + RA 50	HFD+ RA 100	HFD + ME 100
n=1	1.280585177	1.300687444	2.105980646	5.406868093	2.601855915
n=2	0.719414823	2.627819929	3.420815519	5.000455838	1.610274441
n=3	0.967308176	2.960656387	2.293710031	4.131798186	2.415364961
n=4	1.032691824	2.667606418	4.060064982	6.628896751	3.918921426
n=5		2.061234941	3.43887663	5.7305385	4.252028726
n=6		2.363249317	4.225540775	4.055484735	3.793752204
**Mean**	**1**	**2.33**	**3.257**	**5.159**	**3.099**
**Std. Deviation**	**0.2306**	**0.5888**	**0.8827**	**0.9846**	**1.041**

<Table S7: Intensity ratios of Western blots for Figure 4C>

	Group	ABCG5	β-actin	ABCG5/β-actin	fold of control
ABCG5	Ave. of ND-1&2			0.853973742	1
	ND-1	17525.305	20421.548	0.858177108	1.004922126
	ND-2	17182.719	20220.426	0.849770376	0.995077874
	HFD-1	6773.305	19011.134	0.356280956	0.417203643
	HFD-2	4900.841	18172.255	0.269688104	0.315803743
	HFD-3	5367.719	17969.134	0.298718848	0.349798633
	HFD + RA 50-1	12394.012	19242.548	0.644094119	0.754231761
	HFD + RA 50-2	9160.305	19499.033	0.469782527	0.550113551
	HFD + RA 50-3	15041.548	18938.326	0.794238519	0.930050282
	HFD + RA 100-1	13681.962	19260.255	0.710372838	0.831843888
	HFD + RA 100-2	15574.962	19461.841	0.800282049	0.937127233
	HFD + RA 100-3	16032.426	18663.326	0.859033701	1.005925193
	HFD + ME 100-1	5725.598	18038.74	0.31740565	0.371680808
	HFD + ME 100-2	4132.891	15688.912	0.263427509	0.308472609
	HFD + ME 100-3	10173.598	17915.012	0.567881171	0.664986689
	Ave. of ND-3&4			0.793541182	1
	ND-3	17588.719	18607.154	0.945266482	1.191200285
	ND-4	11330.891	17654.426	0.641815882	0.808799715
	HFD-4	8827.598	20602.134	0.428479788	0.539959106
	HFD-5	8523.134	18803.134	0.453282628	0.571215002
	HFD-6	4469.477	19568.134	0.228405887	0.28783117
	HFD + RA 50-4	13381.426	17702.355	0.755912194	0.952580925
	HFD + RA 50-5	12875.255	22540.255	0.571211595	0.719826025
	HFD + RA 50-6	10002.841	19877.548	0.503223084	0.634148668

	**HFD + RA 100-4**	9265.305	16423.548	0.564147589	0.710924148
	**HFD + RA 100-5**	14264.134	16734.447	0.852381558	1.074149114
	**HFD + RA 100-6**	17356.426	18221.376	0.952531027	1.200354876
	**HFD + ME 100-4**	7468.962	16462.205	0.45370362	0.571745525
	**HFD + ME 100-5**	3995.77	15685.355	0.25474527	0.321023377
	**HFD + ME 100-6**	4026.477	15138.305	0.265979381	0.335180313

<Table S8: Summary of relative protein levels of Figure 4C>

**ABCG5/β-actin**	**ND**	**HFD**	**HFD + RA 50**	**HFD+ RA 100**	**HFD + ME 100**
n=1	1.004922126	0.417203643	0.754231761	0.831843888	0.371680808
n=2	0.995077874	0.315803743	0.550113551	0.937127233	0.308472609
n=3	1.191200285	0.349798633	0.930050282	1.005925193	0.664986689
n=4	0.808799715	0.539959106	0.952580925	0.710924148	0.571745525
n=5		0.571215002	0.719826025	1.074149114	0.321023377
n=6		0.28783117	0.634148668	1.200354876	0.335180313
**Mean**	**1**	**0.4136**	**0.7568**	**0.9601**	**0.4288**
**Std. Deviation**	**0.1562**	**0.1186**	**0.1597**	**0.1743**	**0.1512**

<Table S9: Intensity ratios of Western blots for Figure 4D>

	Group	ABCG8	β-actin	ABCG8/β-actin	fold of control
ABCG8	Ave. of ND-1&2			0.287412796	1
	ND-1	3372.335	16429.225	0.205264399	0.714179751
	ND-2	5971.77	16159.083	0.369561194	1.285820249
	HFD-1	3180.062	16374.77	0.194204987	0.675700559
	HFD-2	5241.426	16067.184	0.32621933	1.135020201
	HFD-3	4459.891	17401.426	0.25629457	0.891729852
	HFD + RA 50-1	6710.134	18015.79	0.372458493	1.295900872
	HFD + RA 50-2	6775.962	16266.376	0.416562484	1.449352603
	HFD + RA 50-3	6933.355	17201.154	0.403074991	1.402425349
	HFD + RA 100-1	14663.841	17744.326	0.826396055	2.875293185
	HFD + RA 100-2	9392.184	15273.033	0.614952119	2.139612871
	HFD + RA 100-3	11244.841	16900.326	0.665362372	2.315006084
	HFD + ME 100-1	13661.598	17744.326	0.769913605	2.678772882
	HFD + ME 100-2	18213.719	19773.033	0.921139362	3.204935109
	HFD + ME 100-3	7957.891	17900.326	0.44456682	1.54678854
	Ave. of ND-3&4			0.30698595	1
	ND-3	3113.627	16596.548	0.187606905	0.611125378
	ND-4	6316.426	14814.598	0.426364995	1.388874622
	HFD-4	2588.477	17714.184	0.146124541	0.475997487
	HFD-5	4533.012	11760.77	0.385434967	1.255545954
	HFD-6	4542.134	14077.426	0.322653729	1.051037449
	HFD + RA 50-4	3811.062	12840.719	0.296795063	0.966803409
	HFD + RA 50-5	8297.426	15838.497	0.523877108	1.706518191
	HFD + RA 50-6	4166.477	19337.205	0.215464282	0.701870175

	**HFD + RA 100-4**	6408.719	15647.305	0.409573342	1.33417618
	**HFD + RA 100-5**	10542.598	15168.77	0.695019965	2.264012297
	**HFD + RA 100-6**	18184.891	19017.184	0.956234688	3.114913527
	**HFD + ME 100-4**	11524.719	17289.305	0.666580814	2.171372385
	**HFD + ME 100-5**	15481.548	18773.82	0.824634944	2.686230247
	**HFD + ME 100-6**	17491.255	22007.326	0.79479238	2.589018749

<Table S10: Summary of relative protein levels of Figure 4D>

ABCG8/β-actin	ND	HFD	HFD + RA 50	HFD+ RA 100	HFD + ME 100
n=1	0.714179751	0.675700559	1.295900872	2.875293185	2.678772882
n=2	1.285820249	1.135020201	1.449352603	2.139612871	3.204935109
n=3	0.611125378	0.891729852	1.402425349	2.315006084	1.54678854
n=4	1.388874622	0.475997487	0.966803409	1.33417618	2.171372385
n=5		1.255545954	1.706518191	2.264012297	2.686230247
n=6		1.051037449	0.701870175	3.114913527	2.589018749
**Mean**	**1**	**0.9142**	**1.254**	**2.341**	**2.48**
**Std. Deviation**	**0.3941**	**0.2944**	**0.3617**	**0.6237**	**0.5631**

<Table S11: Intensity ratios of Western blots for Figure 4F>

	Group	CYP7A1	β-actin	CYP7A1/β-actin	fold of control
CYP7A1	Ave. of ND-1&2			0.32099641	1
	ND-1	6498.305	20421.548	0.318208247	0.991314037
	ND-2	6547.062	20220.426	0.323784573	1.008685963
	HFD-1	6547.062	19011.134	0.344380404	1.072848146
	HFD-2	5624.012	18172.255	0.309483441	0.96413365
	HFD-3	3216.648	17969.134	0.179009628	0.55766863
	HFD + RA 50-1	12499.134	19242.548	0.649557117	2.023565049
	HFD + RA 50-2	12582.184	19499.033	0.645272204	2.010216262
	HFD + RA 50-3	9073.77	18938.326	0.479122072	1.492608817
	HFD + RA 100-1	17081.255	19260.255	0.886865465	2.762851659
	HFD + RA 100-2	17119.841	19461.841	0.87966195	2.740410552
	HFD + RA 100-3	13494.426	18663.326	0.723045078	2.252502067
	HFD + ME 100-1	10990.598	18038.74	0.609277477	1.898081903
	HFD + ME 100-2	6581.012	15688.912	0.419468985	1.306771578
	HFD + ME 100-3	10472.719	17915.012	0.584577839	1.821135129
	Ave. of ND-3&4			0.367415404	1
	ND-3	5595.941	16122.305	0.347093111	0.944688512
	ND-4	6136.134	15825.477	0.387737697	1.055311488
	HFD-4	5906.598	16644.891	0.354859518	0.965826458
	HFD-5	10567.134	17516.598	0.603264058	1.641912811
	HFD-6	9770.548	18280.598	0.534476389	1.454692381
	HFD + RA 50-4	11569.255	18159.598	0.637087616	1.733970894
	HFD + RA 50-5	14532.669	17073.305	0.85119249	2.316703332
	HFD + RA 50-6	12882.305	19669.012	0.654954352	1.782599055

	**HFD + RA 100-4**	9283.012	10217.598	0.908531731	2.472764402
	**HFD + RA 100-5**	17920.305	16607.891	1.07902352	2.936794452
	**HFD + RA 100-6**	21180.255	18046.134	1.17367271	3.19440257
	**HFD + ME 100-4**	14242.719	16762.619	0.849671462	2.312563528
	**HFD + ME 100-5**	14025.548	15660.033	0.895626976	2.437641336
	**HFD + ME 100-6**	14280.113	17813.619	0.801640195	2.181836107

<Table S12: Summary of relative protein levels of Figure 4F>

CYP7A1/β-actin	ND	HFD	HFD + RA 50	HFD+ RA 100	HFD + ME 100
n=1	0.991314037	1.072848146	2.023565049	2.762851659	1.898081903
n=2	1.008685963	0.96413365	2.010216262	2.740410552	1.306771578
n=3	0.944688512	0.55766863	1.492608817	2.252502067	1.821135129
n=4	1.055311488	0.965826458	1.733970894	2.472764402	2.312563528
n=5		1.641912811	2.316703332	2.936794452	2.437641336
n=6		1.454692381	1.782599055	3.19440257	2.181836107
**Mean**	**1**	**1.11**	**1.893**	**2.727**	**1.993**
**Std. Deviation**	**0.04572**	**0.3874**	**0.2857**	**0.3328**	**0.4111**

<Table S13: Intensity ratios of Western blots for Figure 5B>

	Group	pAMPKα	AMPKα	pAMPKα/AMPKα	fold of control
pAMPK	Ave. of ND-1&2			0.912595028	1
	ND-1	14751.719	15863.184	0.929934306	1.01899997
	ND-2	13459.548	15034.305	0.89525575	0.98100003
	HFD-1	3756.477	16796.305	0.223649011	0.245069285
	HFD-2	6476.841	16086.012	0.402638081	0.441201265
	HFD-3	5857.305	15884.012	0.368754758	0.404072723
	HFD + RA 50-1	8836.477	14075.184	0.627805434	0.687934313
	HFD + RA 50-2	16297.548	17679.184	0.92184956	1.010140897
	HFD + RA 50-3	14962.012	18748.669	0.798030623	0.874463041
	HFD + RA 100-1	14877.184	12425.426	1.197317822	1.311992489
	HFD + RA 100-2	14413.598	15899.012	0.90657193	0.993400032
	HFD + RA 100-3	17098.548	14521.134	1.177493989	1.290270003
	HFD + ME 100-1	8250.598	11862.477	0.695520674	0.762135069
	HFD + ME 100-2	17431.255	16384.891	1.063861517	1.165754234
	HFD + ME 100-3	17751.012	15436.134	1.149964881	1.260104259
	Ave. of ND-3&4			0.885696849	1
	ND-3	13315.305	13317.184	0.999858904	1.12889518
	ND-4	6531.184	8465.184	0.771534795	0.87110482
	HFD-4	2898.012	14914.184	0.194312475	0.219389371
	HFD-5	6593.841	15223.77	0.433127997	0.489025107
	HFD-6	7387.134	14263.477	0.517905557	0.584743592
	HFD + RA 50-4	9496.598	17223.548	0.551372923	0.622530071
	HFD + RA 50-5	15162.255	19151.548	0.791698666	0.893870929
	HFD + RA 50-6	11040.134	20563.376	0.536883341	0.606170543

	**HFD + RA 100-4**	10209.134	17481.891	0.583983392	0.659349067
	**HFD + RA 100-5**	17561.962	15558.426	1.12877498	1.274448454
	**HFD + RA 100-6**	16893.669	18399.841	0.918142119	1.036632477
	**HFD + ME 100-4**	8210	13625.255	0.60255753	0.680320282
	**HFD + ME 100-5**	18758.962	15546.77	1.20661475	1.362333795
	**HFD + ME 100-6**	16659.598	12798.355	1.301698382	1.469688396

<Table S14: Summary of relative protein levels of Figure 5B>

pAMPKα/AMPKα	ND	HFD	HFD + RA 50	HFD+ RA 100	HFD + ME 100
n=1	1.01899997	0.245069285	0.687934313	1.311992489	0.762135069
n=2	0.98100003	0.441201265	1.010140897	0.993400032	1.165754234
n=3	1.128895	0.404072723	0.874463041	1.290270003	1.260104259
n=4	0.871105	0.219389371	0.622530071	0.659349067	0.680320282
n=5		0.489025107	0.893870929	1.274448454	1.362333795
n=6		0.584743592	0.606170543	1.036632477	1.469688396
**Mean**	**1**	**0.3973**	**0.7825**	**1.094**	**1.117**
**Std. Deviation**	**0.1064**	**0.1417**	**0.1663**	**0.2534**	**0.3237**

<Table S15: Intensity ratios of Western blots for Figure 5D>

	Group	CPT1A	β-actin	CPT1A/β-actin	fold of control
CPT1A	Ave. of ND-1&2			0.301682851	1
	ND-1	3479.598	11083.548	0.313942611	1.040637908
	ND-2	3927.355	13569.598	0.289423091	0.959362092
	HFD-1	4970.062	14061.305	0.353456667	1.171616702
	HFD-2	5886.062	14334.062	0.410634613	1.361146685
	HFD-3	7190.184	18768.184	0.38310494	1.269892996
	HFD + RA 50-1	8809.012	18995.941	0.463731278	1.537148288
	HFD + RA 50-2	9394.234	19057.548	0.49294033	1.633968679
	HFD + RA 50-3	13073.548	21672.891	0.603221232	1.999521117
	HFD + RA 100-1	13209.426	18070.841	0.730980146	2.423008612
	HFD + RA 100-2	15994.255	19071.841	0.838631939	2.779846238
	HFD + RA 100-3	16766.184	18014.062	0.930727562	3.085119217
	HFD + ME 100-1	9239.062	18876.426	0.489449751	1.622398319
	HFD + ME 100-2	17868.598	18527.477	0.964437738	3.196859661
	HFD + ME 100-3	18574.426	18263.477	1.017025728	3.371175141
	Ave. of ND-3&4			0.309472812	1
	ND-3	4880.062	15472.912	0.315393896	1.01913281
	ND-4	4793.426	15791.134	0.303551727	0.98086719
	HFD-4	3874.891	16714.376	0.231829833	0.749112117
	HFD-5	4455.305	17172.962	0.25943719	0.838319813
	HFD-6	5341.598	17557.255	0.304238789	0.983087294
	HFD + RA 50-4	11074.548	17150.669	0.64572105	2.086519481
	HFD + RA 50-5	9334.598	19703.518	0.47375286	1.530838387
	HFD + RA 50-6	13646.134	20685.347	0.659700512	2.131691337

	**HFD + RA 100-4**	13188.012	17823.347	0.739929038	2.390933905
	**HFD + RA 100-5**	17553.134	17805.347	0.985834985	3.185530193
	**HFD + RA 100-6**	10600.477	16254.397	0.652160582	2.107327549
	**HFD + ME 100-4**	10501.849	14270.447	0.735915911	2.37796628
	**HFD + ME 100-5**	16241.255	18315.326	0.886757626	2.86538136
	**HFD + ME 100-6**	12669.77	17760.669	0.713361079	2.305084815

<Table S16: Summary of relative protein levels of Figure 5D>

CPT1A/β-actin	ND	HFD	HFD + RA 50	HFD+ RA 100	HFD + ME 100
n=1	1.040637908	1.171616702	1.537148288	2.423008612	1.622398319
n=2	0.959362092	1.361146685	1.633968679	2.779846238	3.196859661
n=3	1.01913281	1.269892996	1.999521117	3.085119217	3.371175141
n=4	0.98086719	0.749112117	2.086519481	2.390933905	2.37796628
n=5		0.838319813	1.530838387	3.185530193	2.86538136
n=6		0.983087294	2.131691337	2.107327549	2.305084815
**Mean**	**1**	**1.062**	**1.82**	**2.662**	**2.623**
**Std. Deviation**	**0.03667**	**0.2445**	**0.2824**	**0.4255**	**0.6496**
